# Evolution and Treatment of Academic Burnout in Nursing Students: A Systematic Review

**DOI:** 10.3390/healthcare11081081

**Published:** 2023-04-11

**Authors:** José Luis Gómez-Urquiza, Almudena Velando-Soriano, María Begoña Martos-Cabrera, Gustavo R. Cañadas, Luis Albendín-García, Guillermo A. Cañadas-De la Fuente, Raimundo Aguayo-Estremera

**Affiliations:** 1 Ceuta Faculty of Health Sciences, Campus Universitario de Ceuta, University of Granada, 51001 Ceuta, Spain; 2 San Cecilio Clinical University Hospital, Andalusian Health Service, 18016 Granada, Spain; 3 Department of Didactic of Mathematics, Faculty of Education, Campus Universitario de la Cartuja, University of Granada, 18071 Granada, Spain; 4 Casería de Montijo Health Center, Granada Metropolitan District, Andalusian Health Service, 18013 Granada, Spain; 5 Instituto de Investigación Biosanitaria (ibs.GRANADA), 18012 Granada, Spain; 6 Faculty of Health Sciences, University of Granada, Avda. Ilustración 60, 18016 Granada, Spain; 7 Brain, Mind and Behaviour Research Center (CIMCYC), University of Granada, 18071 Granada, Spain; 8 Departamento de Psicobiología y Metodología de las Ciencias del Comportamiento, Facultad de Psicología, Universidad Complutense de Madrid, 28223 Pozuelo de Alarcón, Spain

**Keywords:** burnout syndrome, nursing students, evolution, treatment

## Abstract

Aim: To analyse the scientific literature related to the evolution of burnout syndrome during nursing studies and the interventions for the treatment or prevention of this syndrome in nursing students. Methods: A systematic review of the PubMed, Scopus, and CINAHL databases was performed in August 2022 using the search phrase “burnout AND nursing students” to extract experimental and longitudinal studies. Results: Eleven relevant studies were obtained for analysis. Four were experimental, and seven were cohort studies. According to these studies, the interventions reduced burnout overall, but on occasion, the burnout scores for some aspects increased, as did the prevalence. Psychological and work environment-related variables were the most important factors predicting burnout. Conclusion: Burnout (i.e., emotional exhaustion and depersonalisation) tends to increase during nursing studies. Related factors include personality, coping strategies, life satisfaction, and the work environment. Interventions such as progressive muscle relaxation, behavioural therapy, and recreational music may alleviate burnout.

## 1. Introduction

Burnout syndrome, which has been included in the WHO International Classification of Diseases since 2019, is a consequence of prolonged exposure to chronic stressors in the work environment. Maslach and Jackson [1] described it as a syndrome with three dimensions: emotional exhaustion (the sensation of physical overexertion and emotional weariness provoked by continual interaction with co-workers and/or customers; another description indicates that it is as a chronic indication of emotional and somatic depletion due to workload and personal demands, as well as continuous tension from jobs); depersonalisation (cynical attitudes and unfeeling or impersonal responses towards patients or coworkers); and low levels of personal accomplishment (a loss of confidence in personal fulfilment and the presence of a negative self-concept) [1,2].

Many population groups are susceptible to burnout syndrome (teachers, police officers, etc.), but health professionals (due to the characteristics of their jobs) in general and nurses in particular are amongst the most often affected [3,4]. Burnout may be suffered not only by experienced nurses but also by those students in training to become nurses. The university environment can be highly demanding for student nurses, who are exposed to various stress-producing situations [5]. University entrance, from the outset, provokes important changes. Students often move to another city, develop an independent life in a new environment, undergo changes in their relationships, and are increasingly concerned about the direction of their lives and the outlook for a future career [6,7].

This pressure suffered by students is known as academic burnout. Academic burnout is a variant that overloads students, forcing them to face too many tasks during their training at university. The pressure of having to be an exemplary student who must successfully complete tasks produces stress, guilt, and emotional exhaustion. These feelings can affect learning ability and academic performance [8]. In the case of nursing students, the risk is even greater since they have overexerted themselves during the COVID-19 pandemic [9].

Nursing students are subject to a high prevalence of burnout syndrome, with 73.5% reporting emotional exhaustion, 70.56% depersonalisation, and 76% low levels of personal accomplishment [10]. The consequences of academic burnout may be behavioural (alcohol and drug abuse, voluntary absences from classes, poor diet, or inability to relax, among others), psychosomatic (cardiovascular problems, gastrointestinal disorders, lack of sleep, or chronic fatigue), or emotional (impatience, desire to abandon university studies, depression, lack of self-esteem, or demotivation) [11,12].

Studies of interventions aimed at preventing or reducing burnout in university students have observed positive results from cognitive interventions related to stressors, irrational beliefs, or negative thoughts [12,13], from regular aerobic exercise (to alleviate emotional exhaustion) [14]; from the use of simulated patients and peer group discussions to comment on clinical cases that are especially sensitive, interesting, or shocking to develop empathy [7,15,16,17]; and from mindfulness-based interventions [18,19,20]. On the other hand, the role of nurse educators should be emphasized, as they can intervene to help nursing students to cope with burnout [21]. Recent systematic reviews about burnout have been focused on nursing professionals [4] or medical students [20] but, to our knowledge, not exclusively on nursing students. Each university degree has its own theoretical and practical characteristics, demands, and need for contact with people or with people with a disease process. Thus, nursing students’ burnout should be evaluated on an individual basis.

In light of the negative consequences of burnout in students, it is important to prevent future nurses from entering the labour market suffering from this syndrome and avoid its impact on their care, the patients, and hospitals. Such an outcome would inevitably be detrimental to nurses’ professional development, and it would diminish the quality of care. Therefore, the objectives of this study were: (1) to review the scientific literature related to the evolution of burnout syndrome during nursing studies; and (2) to examine interventions aimed at facilitating the treatment of burnout syndrome or preventing its appearance among nursing students. The research questions were: What is the evolution of burnout during nursing studies, and how effective are the interventions to reduce or prevent it?

## 2. Materials and Methods

### 2.1. Design and Search Strategy

This systematic review is presented in accordance with the Preferred Reporting Items for Systematic Reviews and Meta Analysis (PRISMA) guidelines [22]. The following health sciences databases were consulted: PubMed, Scopus, and CINAHL. The Medical Subject Heading terms employed in the search strategy were “burnout AND nursing students”. This short and general search phrase was used to find as many studies as possible. No filter of the results was applied. The search was conducted in August 2022. The study was registered (ID: 331782) in the PROSPERO database (International Prospective Register of Systematic Reviews).

### 2.2. Study Selection

Eligibility criteria: The data search targeted primary experimental or longitudinal studies about burnout in nursing students published in English, Spanish, or Portuguese. No restrictions were placed on the year of publication (because, to our knowledge, there were no previous reviews) or the burnout questionnaire used. However, studies with mixed samples of university students lacking independent information about nursing students in particular were excluded.

Selection process: The studies were selected for analysis in a four-stage process. First, using a citation manager (Zotero), duplicate studies between databases were deleted. Then, in every case, the title and abstract were read for eligibility, followed by a full-text reading when appropriate. Finally, a critical assessment was performed of the studies remaining, and an inverse search was conducted of the papers cited therein. All these steps were performed independently by two members of the research team, who consulted a third member of the research team if any disagreement arose.

Data collection process and variables: An information table for the data from each study was created. For each study selected, the following data were extracted: authors, year of publication, country of the study, type of study/study design, sample size and characteristics (mean age and % of women), questionnaire used to measure burnout, scores and prevalence of burnout and its dimensions, related factors for its evolution, and information about interventions for burnout prevention and treatment.

Risk of bias, grade of evidence, and data analysis: To determine the risk of bias in the intervention studies considered, the Cochrane risk of bias questions were applied. For longitudinal studies, the CASPe Critical Appraisal Skills Checklist was used. The grade of evidence and level of recommendation were classified according to the guidelines of the Oxford Centre for Evidence Based Medicine [23]. It was not possible to perform a meta-analysis due to the insufficient data available.

## 3. Results

### 3.1. Study Selection Process and Studies Characteristics 

**Figure 1:**
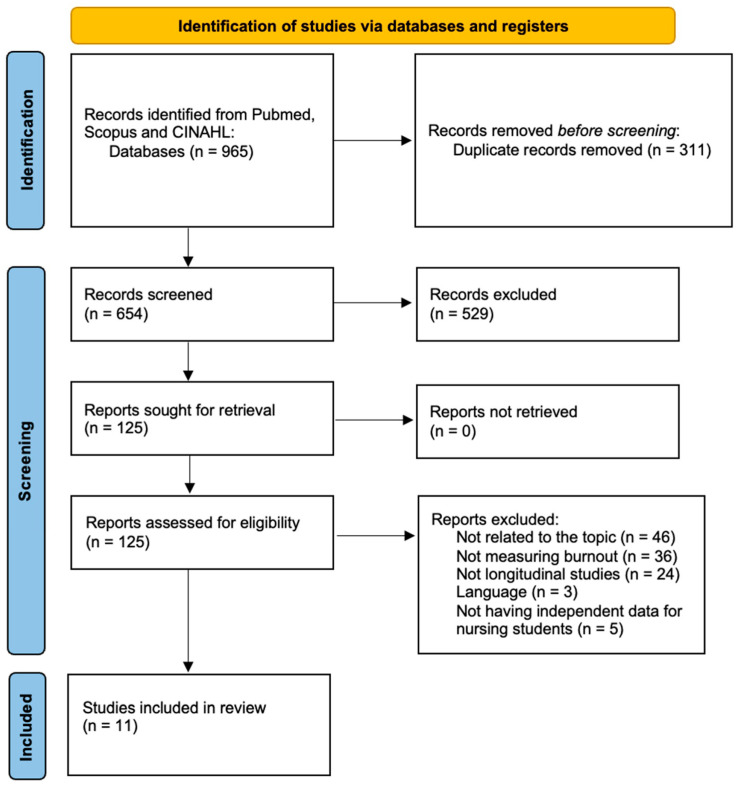
Flow diagram of study selection process.

The search located 964 studies, which were reduced to 654 after eliminating duplicates. After reading the titles and abstracts, 125 remained, and the full text reading produced a final sample of *n* = 11 studies [24,25,26,27,28,29,30,31,32,33,34]. The study selection process is illustrated in Figure 1. 

Of these 11 papers, seven referred to cohort studies, three to clinical trials, and one to a quasi-experimental study. By geographic location, 77.73% of the studies took place in Europe, 18.18% in the United States, and 9.09% in Asia. By date of publication, 45.45% of the studies were published after 2015, with the oldest from 1988 and the most recent from 2020. The largest sample from the included studies was *n* = 1702 nursing students, and the lowest was *n* = 75 nursing students. All the population samples analysed had a majority of female participants. The highest evidence level was A, and the lowest was B, while the highest grade of recommendation was 1b, and the lowest was 2b. The characteristics of the studies are detailed in Table 1.

### 3.2. Evolution of Burnout and Related Factors during Nursing Studies

Among the longitudinal studies considered, one reported correlations between burnout and low-complexity self-efficacy psychomotor skills (from −0.12 to −0.22) and between burnout and moderate-complexity self-efficacy (from −0.07 to −0.21), correlations that persisted throughout the course of nursing studies [28]. Another study showed that dispositional variables (such as life satisfaction or the absence of hobbies) and experiences (for example, the working environment and conditions) are useful predictors of burnout scores, with R values of 0.70 for emotional exhaustion, 0.69 for depersonalisation, and 0.60 for personal accomplishment according to dispositional variables and similar R values according to experiences [29]. However, the analysis of the differences in burnout during nursing studies did not show any pattern [29].

One study [30] observed that personal accomplishment scores significantly increased during nursing studies and that personality traits and emotion-oriented coping were correlated with certain variables (emotional exhaustion and depersonalisation) but not all the measures considered, while another study [31] found that emotional exhaustion and depersonalisation were correlated with neuroticism for all measures, including emotional and behavioural coping. One author reported that scores for emotional exhaustion and depersonalisation remained stable during nursing studies, but those for personal accomplishment fell significantly [30]. However, another study [32] found that emotional exhaustion increased significantly during nursing studies, while depersonalisation and personal accomplishment did not change. Finally, two studies [33,34] indicated that emotional exhaustion and depersonalisation increased during nursing studies, as did the overall prevalence of burnout (from 29.7 to 41%). The characteristics and results of these studies are summarised in Table 1.

### 3.3. Interventions to Alleviate Burnout in Nursing Students

According to the studies examined, interventions to alleviate burnout showed positive results [23,24,25]. One study used recreational music and obtained significant reductions in emotional exhaustion and depersonalisation after six weeks, with one session per week, reducing emotional exhaustion by more than 2 points and depersonalisation by 1.3 points [24]. In another study, a 30-min progressive muscle relaxation programme four times per week was applied every night before sleeping for three weeks; this programme also achieved a significant reduction in burnout scores of 1.13 points with respect to the control group (only a 0.51-point reduction) [25].

Six sessions of behaviour therapy for two hours was also reported to reduce burnout in nursing students, obtaining significantly lower levels of burnout than in a control group (2.1 vs 2.8), at least immediately following the intervention [26]. However, after three months, this difference in burnout levels had disappeared. In contrast to the above positive results, a year-long internship programme for nursing students resulted in an increase in burnout prevalence, from 34.7 to 43.6% [27]. Details of these studies are shown in Table 1.

## 4. Discussion

The aim of this systematic review is to summarise and analyse study findings on the evolution of burnout among nursing students during their studies and the effectiveness of interventions to reduce or to prevent this syndrome. Burnout tends to increase during nursing studies. Some variables are correlated with its appearance, while interventions based on music, relaxation, or behavioural therapy may be useful for reducing its impact.

The studies selected for analysis show that burnout, especially in the components of emotional exhaustion and depersonalisation, tends to worsen during nursing studies. A similar trend has been identified among medical students [35,36]. As students become more advanced, they are exposed to greater clinical responsibilities, coming into contact with patients during their practical experience and facing complex situations for which they may not feel prepared [37]. Contact with relatives and patients, relationships with hospital professionals, and the perceived need to protect oneself from emotionally complex situations [38] can all aggravate emotional exhaustion and depersonalisation. 

Some studies have shown that personality factors, such as neuroticism, are related to burnout in nursing students and also in oncology students and primary care nurses [39,40]. In this respect, it has been observed that persons with neuroticism tend to experience negative emotions in response to threats, frustration, or loss [41]. For nursing students, the university environment, together with the associated clinical placements, puts them in complex situations that can create feelings of anxiety, frustration, and loss or that may be perceived as threatening (in areas such as exams, scholarships, and feelings of insecurity in dealing with patients) [42,43]. All of these pressures contribute to burnout in students. Burnout may also be aggravated by certain coping and work environment variables. The ability to cope with difficult situations is a quality of great importance. For example, nursing students may have manage situations such as the COVID-19 pandemic, for which effective coping strategies are needed [44]. Occupational risk factors are known to heighten burnout in nurses; therefore, a good work environment will help to prevent its appearance among nursing students [45]. Additionally, while some studies have identified differences in burnout according to gender [46], the studies included in this review did not analyse these factor. Systematic reviews with meta-analysis have focused on nursing professionals [4,45,46] or medical students [20], but the different characteristics of a nursing degree make necessary this review’s focus on nursing students.

Interventions such as muscle relaxation, music, behavioural therapy, and mindfulness have shown some effectiveness in reducing and preventing burnout among nurses [47,48,49]. However, this impact needs to be confirmed in further investigations. More studies in the future should analyse the effectiveness of interventions for reducing and preventing burnout in nursing students, as has been undertaken in nursing professionals. 

### Limitations

The present study has some limitations. First, it was not possible to perform a meta-analysis because the interventions differed in scale and type, and to date, very few clinical trials have been conducted of burnout treatment programmes for nursing students. Moreover, the studies identified were performed in several different countries, so the conditions to which these students were exposed were not homogeneous. Accordingly, any generalisation of these study results should be considered with caution. Future research in this field should be experimental, investigating interventions in areas such as mindfulness, sport, or behavioural therapy to prevent or reduce the appearance of burnout syndrome in nursing students and in later professional life. It would also be useful to study the question of burnout in students presenting particular risk factors, such as neuroticism and other personality traits.

With respect to the applicability of the results that we present, the academic managers and teachers of nursing students should promote the use of interventions that have shown benefits in reducing or preventing burnout (such as muscle relaxation and the use of recreational music) and be aware that burnout tends to increase during nursing studies. It would be useful to improve students’ conditions in both the academic and the practical elements of their education and, in particular, to offer greater support during the final stage of the course. Finally, nursing students need to be taught the importance of good rest. Interspersing leisure time and obtaining the necessary hours of sleep are essential to better cope with the pressure to which nursing students are exposed.

## 5. Conclusions

The prevalence of burnout tends to increase during nursing studies, particularly regarding emotional exhaustion and depersonalisation. Certain personality factors (neuroticism) are correlated with the appearance of burnout in these students. Other variables that might influence burnout in nursing students include emotional and behavioural coping. Interventions based on recreational music and programmes to achieve muscle relaxation may contribute to reducing burnout. Dispositional variables (such as life satisfaction or the absence of hobbies) and experiences (such as the working environment and conditions) could help to predict burnout scores.

## Figures and Tables

**Table 1:** Characteristic of included studies.

Authors, Year of Publication, and Country	Type of Study	Sample	Intervention or Follow up Time	Results	EL and GR
Ayaz-Alkaya et al., 2018. Turkey [27]	Quasi-experimental study	*n* = 101. 87.1% of the sample was female.	Nursing internship programme (two courses with 3 h of theory and 18 practical training per week for one academic year). Training was about internal medicine, general surgery, gynaecology, paediatric, psychiatry, and public health clinics. Students were responsible for caring (under supervision) for patients.	Burnout prevalence was significantly increased after the internship, from 34.7% to 43.6%. Additionally, students with dangerous signs of burnout or requiring help were increased.	B/2b
Bittman et al., 2004. United States [24]	Crossover clinical trial	*n* = 75. 85.3% of the sample was female. The mean age was 27.5.	Six-week intervention with one session per week about recreational music. The HealthRHYTHMS Group Empowerment Drumming Protocol^®^ was followed with instruments such as hand drums, SoundShapes^®^, and percussion auxiliary instruments. Control group continued its academic routines.	EE (25.9 to 23.2 points) and D (6.4 to 5.1 points) showed a significant reduction after the intervention.	A/1b
Bulfone et al., 2016. Italy [28]	Quantitative longitudinal study (cohort study)	*n* = 571. 70.6% of the sample was female. The mean age was 22.7.	First measure was at the beginning of the second year, and second measure was at the beginning of the third year.	The correlation of burnout with low-complexity self-efficacy psychomotor skills increased from −0.12 to -0.22, such as middle complexity self-efficacy from −0.07 to −0.21. High complexity self-efficacy did not significantly change.	B/2b
Burisch, 2002. Germany [29]	Quantitative longitudinal study (cohort study)	*n* = 221. 74% of the sample was female. The mean age was 21.4.	First measure during their first week prior to practical training and then after each half-year (seven points of measures).	Dispositional variables (life satisfaction, health concerns, lack of hobbies, addiction, emotional isolation and others, etc.), particularly the “attitudinal” components, helped to predict burnout scores of the three dimensions. R = 0.70 for EE, R = 0.69 for D, and R = 0.60 for PA. The experiences (inventory of nursing stresses, job environment, and conditions, tedium), particularly the “emotional” components during nursing studies, helped to predict the evolution and score of emotional exhaustion at the end of the study. R = 0.70 for EE, R = 0.69 for 0.38 for D, and R = 0.50 for PA. The differences in the burnout scores among the seven measures did not show any pattern.	B/2b
Deary et al., 2003. United Kingdom [30]	Quantitative longitudinal study (cohort study)	*n* = 168 students. 89% of the sample was female. The mean age was 25.4.	First data collection was performed upon entry into nurse education, second collection at 12 months, third collection at 24 months, and fourth collection at the end of the nursing programme.	The burnout scores were stable during the nursing programme. Only the personal accomplishment scores significantly increased during the study. Some personality traits were correlated with EE and D but not all the measures. PA was correlated with conscientiousness over the duration of the study. Emotion-oriented coping was correlated with EE at the second measure only and task-oriented coping with PA only at time 2.	B/2b
Fornés-Vives et al., 2019. Spain [31]	Quantitative longitudinal study (cohort study)	*n* = 249. 88.5% of the sample was female. The mean age was 20.97.	At the beginning of nursing studies and at the end.	EE and D were significantly correlated with neuroticism at both measures. EE was significantly correlated with emotional coping and behavioural coping at both measures. PA was only correlated with emotional coping at the firs measure.	B/2b
Frogeli et al., 2015. Sweden [26]	Randomised controlled pilot trial	*n* = 69 intervention group. *n* = 44 control group. No data about gender or mean age	The intervention consisted of six group sessions of 2-h using behaviour therapy to target stress. It included information about behaviour change strategies, lifestyle factors, communication and assertiveness skills, and training in acceptance and commitment therapy. The control condition consisted of two seminars of 3 h for reflection about personal and professional development.	The intervention group had significantly lower levels of burnout than the control group at post-intervention (2.1 vs 2.8). Cohen’s d = 0.82. The difference was not significant at three months of follow-up.	A/1b
Haack, 1988. United States [32]	Quantitative longitudinal study (cohort study)	*n* = 283 No data about gender or mean age	Data were collected three times during the nursing studies, one measurement per year.	Emotional exhaustion significantly increased during nursing studies. Depersonalisation scores increased, but the differences were not statistically significant. Personal accomplishment scores did not change.	B/2b
Pelit-Aksu et al., 2020. Turkey [25]	Randomised controlled trial.	Control group (*n* = 78) Intervention group (*n* = 67) 84.8% of the sample was female. The mean age was 21.99.	Students saw a demonstration about a progressive muscle relaxation exercise, and then they performed it for 30 minutes. The students tensed and relaxed the muscle groups in the hands, arms, neck, shoulder, chest, stomach, hips, feet, and fingers and all muscles in the face and body, in sequence. During the PMRE, the students listened to the audio instructions and applied the exercises exactly according to these audio instructions. They were told to perform this kind of routine before sleep at least four times per week. They received verbal direction instructions on their phones.	Burnout scores significantly decreased after three weeks in both groups, but the decrease was greater in the intervention group (3.64 to 2.51) than in the control group (3.31 to 3.02). The differences between groups in favour of the experimental group were statistically significant (2.51 vs 3.02)	A/1b
Rudman and Gustavsson, 2012. Sweden [33]	Quantitative longitudinal study (cohort study)	*n* = 1702. 91% of the sample was female. The mean age was 28.	Measurement over the three years of higher education.	Emotional exhaustion and depersonalisation increased over the years in nursing higher education. The increase was greater in D than in EE. The burnout prevalence increased from 29.7% to 36.9% in the second year and 41% in the third year. Changes in EE predicted health problems and lower levels of satisfaction with life. Higher D and its increasing predicted lower levels of engagement and occupational preparedness.	B/2b
Watson et al., 2008. China [34]	Quantitative longitudinal study (cohort study)	*n* = 158. 82.3% of the sample was female. The mean age was 19.1.	Two measurements. At the start of the studies and after seven months.	Higher levels of burnout were reported at the second measurement, which is largely explained by neuroticism and emotion-oriented coping. EE increased from 22.6 to 24.5. D increased from 10.4 to 11.5 PA significantly decreased from 29.1 to 27.	B/2b

Table footnote: D = depersonalisation; EE = emotional exhaustion; EL = evidence level; GR = grade of recommendation; PA = personal accomplishment.

## Data Availability

The datasets generated and/or analysed during the current study are not publicly available due to respondents’ confidentiality but are available from the corresponding author on reasonable request.
